# Heat Transfer Performance of a Novel Multi-Baffle-Type Heat Sink

**DOI:** 10.3390/e20120979

**Published:** 2018-12-17

**Authors:** Xin Cao, Huan-ling Liu, Xiao-dong Shao

**Affiliations:** 1 Key Laboratory of Electronic Equipment Structure Design, Ministry of Education, School of Electromechanical Engineering, Xidian University, Xi’an 710071, China; 2 Department of Mechanical, Aerospace and Nuclear, University of Rensselaer Polytechnic Institute, Troy, NY 12180, USA; 3 Xidian-Ningbo Information Technology Institute, Xidian University, Xi’an 710071, China; 4 Department of Mechanical Engineering, University of Alberta, Edmonton, AB T6G1H9, Canada

**Keywords:** multi-baffle-type, heat transfer, heat sink, friction factor

## Abstract

A new type of multi-baffle-type heat sink is proposed in this paper. The heat-transfer coefficient and pressure drop penalty of the employed six heat sink models are numerically investigated under five different inlet velocities. It is shown that Model 6 (M6) has excellent heat transfer performance as its heat-transfer coefficient reaches a value of 1758.59 W/m^2^K with a pressure drop of 2.96 × 10^4^ Pa, and the temperature difference between the maximum and the minimum temperature of the heating surface is 51.7 K. The results showed that the coolant for M6 is distributed evenly to each channel at the maximal degree. The phenomena of the maldistribution of temperature is effectively improved. Moreover, the thermal resistance and thermal enhancement factor for the six models is also examined. M6 possesses the lowest total thermal resistance and largest thermal enhancement factor compared to the other five models. Furthermore, an experimental platform is set up to verify the simulation results obtained for M6. The simulated heat-transfer coefficient and pressure drop values agree well with the experimental results.

## 1. Introduction

Temperature uniformity is an important factor in the the performance of heat sinks covering many industrial applications in small and large scales [1]. Failures in many industrial facilities are attributed to large stress, localized corrosion, and excessive thermal expansion which resulted from a non-uniform fluid flow, heat or mass transfer, and chemical reactions. Temperature distribution can be a key factor in all of the above mentioned failures. This may happen in any application where a heat source exists. Some fields have been extensively investigated in the literature such as micro-channel heat sinks [2,3], heat exchanger reactors [4,5], plate-type heat exchangers [6,7], and fuel cells [8,9]. In general, uniform flow distribution may provide better heat-transfer performance, low pressure penalty, and a minimization of flow-caused vibrations.

Numerous studies related to the flow uniformity phenomena in heat exchangers have been performed in recent years [10,11,12]. Liu et al. [10] studied the issues of uniform flow distribution for general application in fuel cells, fuel processing chemical reactors, and other industrial devices. Wang et al. [11] examined how to solve the flow and pressure distribution in fuel cell stacks based on plate heat exchangers in a U-type arrangement. Wang et al. [12] optimized the cathode flow field for a single serpentine PEM fuel cell with five channels using the heights of channels 2–5 as search parameters. 2D flow distributors have been adopted, which could achieve flow uniformity for multiple parallel flow channels on a flat plate [13,14,15]. Luo et al. [13] introduced the constructal approach and discussed some examples like coupling of fluid distributor to heat exchanger, and mixer and its coupling to a micro reactor of multi-scale components. Fan et al. [14] experimentally investigated the flow distribution behavior of a plate-type structural flow distributor according to structural theory to achieve uniform flow distribution with the smallest energy dissipation. Liu et al. [15] studied the details of flow in a channel with a fundamental structured symmetric bifurcation of one flow channel into two sub-channels. Brenk et al. [16] made minimum geometrical modifications to the PHE (Multi-Plate Heat Exchangers) based on a robust 2D model which helped reduce flow maldistribution. Liu et al. [17] studied the flow distribution uniformity in flow channels having three major types of generic bifurcation structures. Their results indicated that Tee-type bifurcation is favourable to produce uniform flows. Cao et al. [18] numerically examined the effect of bifurcation angle and inlet velocity on the flow uniformity of consecutive bifurcating fluid flow distributors. They found that the flow distribution uniformity decreases with increasing bifurcation angles from 15° to 90°. Bahiraei et al. [19] did a research on the performance of a novel distributor liquid block and compared the performance with two conventional liquid blocks including serpentine and parallel geometries. They found that both thermal performance and irreversibility rates of the novel distributor liquid block are better than the DC (Direct Channels) channels.

The inlet configurations are regarded as an important factor affecting flow maldistribution. Therefore, different header or manifold designs have been introduced into the heat sink [20,21,22,23,24]. Zhang et al. [25] experimentally investigated the flow distribution performance in plate-fin heat exchangers with new header configurations. It was reported that the degree of the flow and temperature maldistribution in the plate-fin heat exchanger is reduced to 16.8% and 74.8% by changing the header configuration. Cyril et al. [26] studied the improvement of flow distribution uniformity among parallel mini-channels. A relatively uniform flow distribution may be achieved by introducing the discrete stairway shape distributor/collector or by a continuous tapered shape distributor/collector with very low flow rates. Flow distribution performance for different geometric configurations by varying the header shape (rectangular, trapezoidal and triangular) was numerically studied by Kumaran et al. [27], the header size and locations of inlet and outlet arrangement (I, C, V, Z and U-type) have also been studied. Temperature distribution performance was shown to be better for C-type, and a triangular inlet header provides better temperature distribution. Wang et al. [28] experimentally and numerically studied the single-phase flow distribution performance in a parallel flow heat exchanger subject to various operating conditions. They found that flow distribution for U-type flow is more uniform than Z-type flow. Zhang et al. [29] experimentally investigated different distributor configurations in a plate-fin heat exchanger under different operating conditions. They found that an improved distributor design with a complementary fluid cavity is very effective in improving the flow distribution.

In order to solve the maldistribution problem, baffles are sometimes added to headers or channels. Liu et al. [30] numerically investigated the fluid and thermal performances of four mini-channel heat sinks with/without the baffles. They found that the flow uniformity can be improved through the nonuniform mini baffles leading to better uniform temperature distribution. In a numerical study supported by experiments, Ahmed et al. [31] explored the effect of using sub-channels in a liquid cooled heat sink to minimize the effect of hot spots generated on a chip or circuit. It was found that the improvement of the temperature uniformity is reached by adding sub-channels where the maximum temperature is reduced. Wen et al. [32] proposed an improved header configuration with punched baffle which can improve the flow maldistribution in both radial and axial directions. Ismail et al. [33] employed modified headers with porous baffle plates to improve the flow distribution. Dabiri et al. [34] adopted small cylindrical obstacles in a distributor to evaluate the geometry-induced maldistribution effect. Their results indicated that the flow becomes more uniform by applying the obstacles in the distributor. Luo et al. [35] developed a heuristic algorithm for baffled fluid distributor designs. They reported that the developed algorithm can successfully reach a uniform flow distribution with a small increase in pressure drop. Chai et al. [36,37] selected five different configurations of ribs and four lengths along the flow direction for every rib configuration to analyze the effects of rib geometry on heat-transfer characteristics and the thermal-hydraulic performance. They showed that the ribs in the transverse microchambers can effectively prevent the decline of the local heat-transfer coefficient along the flow direction. Then they firstly examined the effects of rib geometry on thermal-hydraulic performance by the variations of friction factor and Nusselt number with Reynolds number. The need to improve the flow distribution is still growing in order to optimize devices performance.

The temperature uniformity of the parallel channel heat sink is a crucial factor for the life time of the heat sink. Until now, no data has been reported about the flow and heat-transfer of the heat sink equipped baffles for improving the velocity uniformity of the parallel channels. Consequently, a novel design fitted with a multi-baffle is proposed to achieve temperature uniformity of the heating surface of the heat sink. Numerical simulations supported with experiments are undertaken to improve the heat-transfer performance and to lower pressure drop. The temperature uniformity of the heating surface and thermal resistance of six different models are numerically examined. The flow and heat-transfer behaviors of the selected geometry are experimentally evaluated, in a laminar regime to verify the simulation results.

## 2. Problem Description

### 2.1. Geometric Configurations and Computational Domain

**Figure 1:**
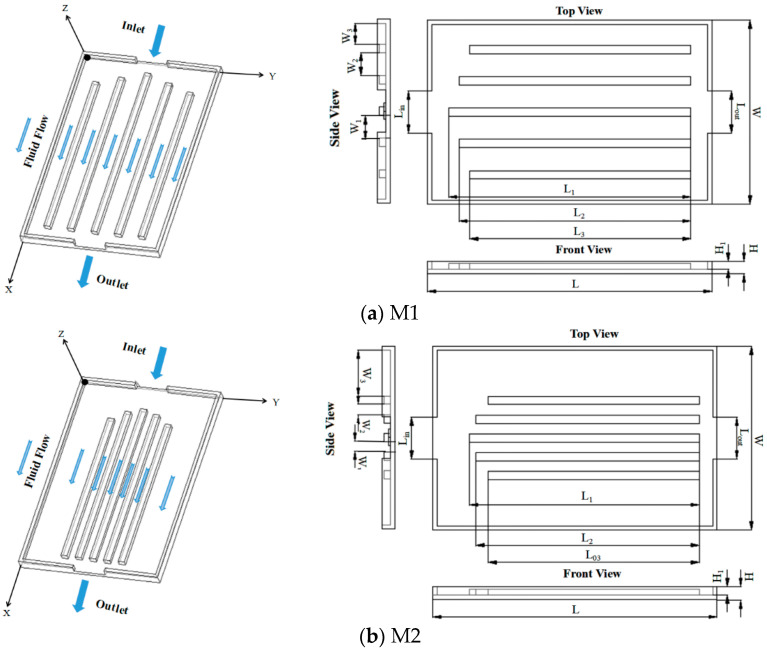

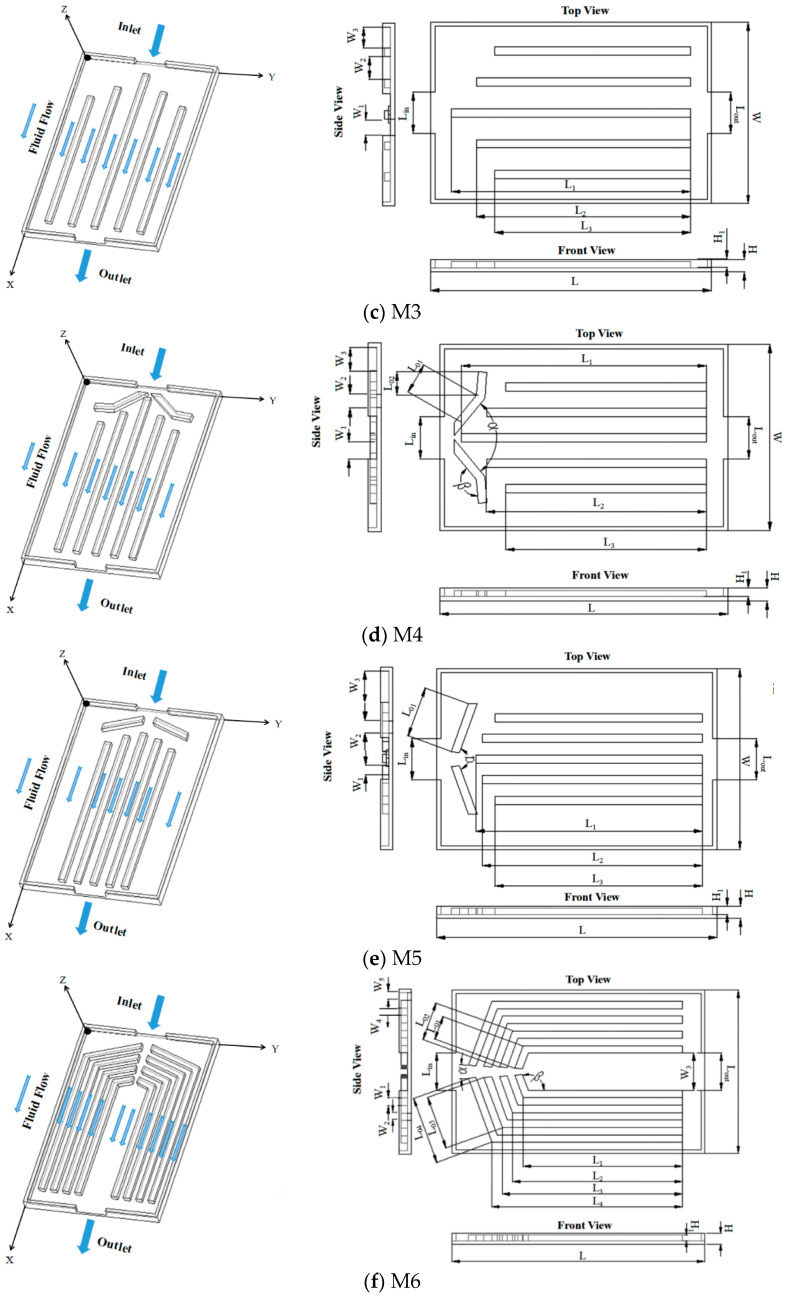
Schematic of heat sinks and geometric configuration.

Six different heat sink configurations (M1 to M6) with dimensions L × W × H and are depicted in Figure 1. Three configurations (M1 to M3) are without baffles and the other configuration are fitted with different baffles (M4 to M6). All the channels and baffles are arranged symmetrically. As shown in the front view of the models, H_1_ is the depth of the counter sink. These configurations are chosen to see the effect of having baffles on the flow and temperature distribution.

As shown in Figure 1a, M1 has five parallel channels of the same width, and the length of the channel at the inlet, excluding the middle channel, is cut down according to the angle between the cut direction and the main flow direction that is 15°.

Based on M1, the width of the channel in M2 is gradually widened such that more coolant can flow into the side channels, and the obtained lengths of the channels are the same as M1, as shown in Figure 1b. For M3, the width of the channels is maintained the same as M1. However, the length of the channel at the inlet, excluding the middle channel, is cut down to 30° between the cut direction and flow direction, and as can be seen in Figure 1c. Based on M2 and M3, a pair of whirl baffles is added to the entrance of M4 and is symmetrically placed along the centre line of the heat sink shown in Figure 1d. The angle between the whirl baffle and the centre line of the heat sink is *α* and the angle between the top and bottom walls of the whirl baffle is *β*. M5 is designed based on M4, while a pair of direct baffles is added to it (Figure 1e). The angle between the direct baffle and the centre line of the heat sink is *α*. M6 configuration comprises nine flow channels, presented in Figure 1f, which are seamlessly connected with whirl baffles. The baffles are described by the Cartesian coordinate system, which are symmetrical in the middle channel on the horizontal surface. The angle between the formed baffle and the centre line of the heat sink is *α* and the angle between the baffle and the wall of the channel is *β*. Detailed parameters of six heat sinks are listed in Table 1. The channels are arranged with different baffles to prevent the maldistribution of the coolant and to achieve temperature uniformity.

### 2.2. Mathematical Model

In the present simulation, the inlet velocity range is 0.06–0.3m/s, and the corresponding ranges of the inlet mass flow rate and Reynolds number (*Re*) are 0.04–0.22 kg/s and 400–2000, respectively. The inlet velocities (*u*_1_) and the corresponding *Re* are inserted in Table 2. It should be noticed that this study is limited to the conditions applied for the simulation and experiment including:

(1) The volume force and the effect of surface tension are all neglected.
(2) No radiation and gravity is assumed.
(3) The thermo-physical properties of water are considered as constant and incompressible.
(4) Axial conduction and viscous dissipation are not considered.

The general forms of the governing equations including continuity, momentum, and energy equations can be expressed as follows.

Continuity equation:

(1) $$\frac{\partial (\rho u_{i})}{\partial x_{i}}=0$$

Momentum equation:

(2) $$\frac{\partial (\rho u_{i}u_{j})}{\partial x_{i}}=\frac{\partial }{\partial x_{i}}(\mu \frac{\partial u_{i}}{\partial x_{i}})-\frac{\partial p}{\partial x_{i}}$$

Energy equation:

(3) $$\frac{\partial (\rho u_{i}T)}{\partial x_{i}}=\frac{\partial }{\partial x_{i}}(\frac{k}{c_{p}}\frac{\partial T}{\partial x_{i}})+S_{T}$$

For the solid:

(4) $$\frac{\partial }{\partial x_{i}}\left(k_{s}\frac{\partial T}{\partial x_{i}}\right)=0$$

ANSYS ICEM CFD 14.5 software is used to generate the unstructured mesh for the solver. The grids at the wall of the inlet, outlet, and six channels are densified. Commercial software Fluent 14.5 as a computational fluid dynamics (CFD) tool is used for simulation in this work. The following boundary conditions are imposed:

(1) The coolant used is water and the multi-baffle-type heat sink (MBHS) is fabricated using aluminum.
(2) A uniform heat flux of *q* = 58000 W/m^2^ is applied to the bottom of heat sinks, and other surfaces are considered to be adiabatic.
(3) The inlet velocity *u*_1_ (Table 2) and is assumed to remain constant. The inlet temperature *T_in_* = 293 K.
(4) The outflow condition in the software is set at the outlet.

### 2.3. Parameter Definition

In order to improve the physical understanding of the simulation, the non-dimensional parameter is defined as

(5) $$Re=\frac{\rho u_{m}D_{h}}{\mu }$$

where *u_m_* is the average water velocity and *µ* is the fluid dynamic viscosity and the *D_h_* is the channel hydraulic diameter.

The flow friction factor *f* is defined as

(6) $$f=\frac{2\Delta PD_{h}}{\rho {u_{m}}^{2}L_{3}}$$

Using the friction factor, the loss of resistance along the tube *h_f_* can be evaluated as

(7) $$h_{f}=\frac{1}{2}fu^{2}\frac{L_{n}}{D_{h}}$$

where *L_n_* is the length of channel and *u* is the fluid velocity.

The pressure drop along the pipe ΔP is expressed as

(8) $$\Delta P=\rho h_{f}$$

The head loss *h_j_* can be evaluated as

(9) $$h_{j}=\zeta \frac{u_{m}^{2}}{2g}$$

and ζ is the head loss coefficient.

The local pressure drop along the pipe $\Delta P^{\prime }$ is expressed as

(10) $$\Delta P^{\prime }=\zeta \frac{\rho u_{m}^{2}}{2}$$

The total thermal resistance of the heat sink is calculated as:

(11) $$T_{total}=\frac{T_{b,avg}-T_{in}}{q}$$

### 2.4. Field Synergy Principle

Guo et al. [38] proposed a method for improving the heat transfer coefficient by analysing the boundary-layer type flow in 1998 known as the field synergy principle. This factor is expressed as an angle between the temperature gradient and velocity vector. The field energy angle can be defined as

(12) $$\theta =\arccos\frac{V\cdot \nabla T}{\left|V\right|\left|\nabla T\right|}$$

This angle is used in this study to improve heat-transfer coefficient. The smaller the field synergy angle between the velocity vectors and temperature vectors, the higher the heat-transfer rate.

## 3. Experiment Apparatus and Procedure

**Figure 2:**
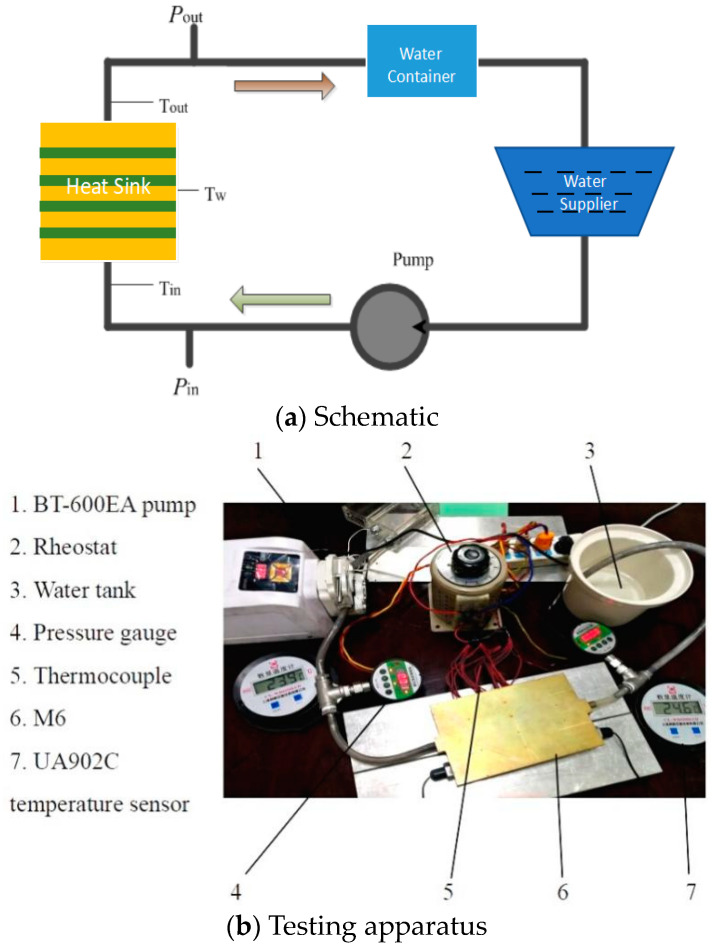
(**a**) Schematic and (**b**) testing apparatus of performance evaluation.

**Figure 3:**
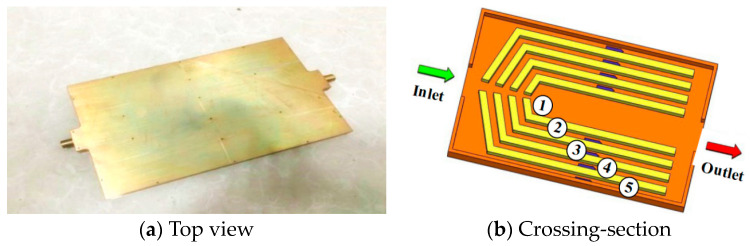
Model employed in experiment and simulation (①–⑤ represents flow channel).

**Figure 4:**
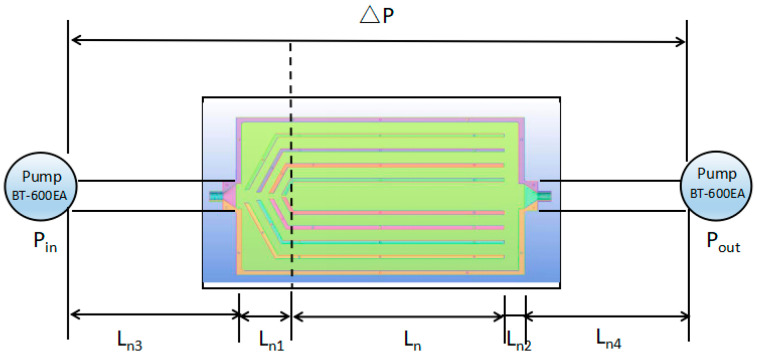
The method of measuring pressure-drop of heat sink.

Figure 2a shows the schematic of the experimental setup used to test the heat-transfer performance of the MBHS. An annotated picture of the experimental apparatus is shown in Figure 2b. Figure 3a shows the top view of the heat sink and Figure 3b shows a drawing of the channel with the locations of the inlet and outlet. Water is selected as the coolant, which is pumped into the channels using a peristaltic pump BT-600EA. When the temperature of the water decreases to the entrance temperature of 293 K, the coolant is flushed into the water container. The inlet and outlet pressures (*P_in_* and *P_out_*) are measured using HG-80K intelligent digital pressure gauges that measures pressures ranging from 0 to 1MPa. The relative accuracy is ±2%. The pressure drop of the MBHS Δ*P* is equal to $\Delta P_{in}-\Delta P_{out}$ as shown in Figure 4. The pressure drop between the inlet and outlet, Δ*P* can be expressed as

(13) $$\Delta P=\Delta P_{Ln3}+\Delta P_{Ln4}+\Delta P_{Ln1}+h_{f_{\mathrm{Ln}1}}+\Delta P_{Ln}+\Delta P_{Ln2}+h_{f_{\mathrm{Ln}2}}$$

where $\Delta P_{Ln3}$ is the pressure drop from the gauge to the inlet port of the heat sink due to frictional resistance, $\Delta P_{Ln4}$ is the pressure drop from the outlet of the heat sink due to the frictional resistance, $\Delta P_{Ln1}$ is the pressure drop from the inlet of the heat sink due to flow contraction, $\Delta P_{Ln}$ is the pressure drop from the inlet parallel channels to the outlet of the parallel channels, $\Delta P_{Ln2}$ is the pressure drop from the outlet of the parallel channels to the outlet of the heat sink due to flow sudden expansion.

To satisfy the constant-heat-flux boundary of *q* = 58000 W/m^2^, five stainless steel heating bars are placed on an aluminum plate. The heating-surface is tightly contacted with the bottom of the heat sink, and the heat flux is transferred to the heat sink from the heating unit. This is controlled by setting the power to each rod to a value between 0 to 300 W using a rheostat. The heat flux input section is 60 × 80 mm for which the corresponding heat flux of the heat sink can reach 0–60,000 W/m^2^.

Based on the law of energy conservation, the heat quantity (*Q*_1_) supplied by electrical heating source should be equal to the sum of that (*Q*_2_) taken away by cooling fluid (water) and the heat loss (*Q_loss_*) into environment:

(14) $$Q_{1}=Pt$$

(15) $$Q_{2}=c_{p}G\left(T_{out}-T_{in}\right)$$

(16) $$Q_{loss}=Q_{1}-Q_{2}$$

Here, *U* is heating electric voltage, *I* is heating electric current, *c_p_* is isobaric specific heat capacity of water, *G* is mass flow rate of water, *T_out_* is outlet water temperature, and *T_in_* is inlet water temperature. So the maximum relative error of heat balance is

(17) $$\frac{\Delta Q_{B}}{Q_{B}}=\frac{\left|Q_{loss}\right|}{\frac{Q_{1}+Q_{2}}{2}}=\frac{\left|Q_{1}-Q_{2}\right|}{\frac{Q_{1}+Q_{2}}{2}}$$

Under steady state of experiments, calculations show that $\Delta Q_{B}/Q_{B}<4.1\%$ when (*T_out_* − *T_in_*) is over 275 K. That is to say the relative error derived from electric heating power insteading of heat quantity flowing into heat sink is not more than 4.1%, which means the heat loss into the surrounding environment is not more than 4.1%. The heating unit is packaged in a layer of insulation material was used over remainder of the heating unit to ensure that 95.9% of the heat was transferred to the heat sink.

To measure the temperature, two K-type thermocouples are placed at the inlet (*T_in_*) and the outlet (*T_out_*). Five thermocouples (*T*_1_, *T*_2_, *T*_3_, *T*_4_, and *T*_5_) are embedded in the bottom of the heating unit in order to measure the temperatures of the heating surface of the heat sink. The temperature at the heating surface (*T_h_*) is considered to be the mean value of the five temperature readings. During the measurement, the peristaltic pump with a fixed mass flow rate of 0.04 kg/s first push the coolant into the channels. The steady state stability is defined as the time of the fluctuation range of date less than 0.1% is below twenty minutes. The fluctuation range of temperature is less than 0.2 K.

The heat-transfer coefficient (h) of the heat sink is

(18) $$h=\frac{q}{\Delta T}=\frac{q}{T_{h}-T_{m}}$$

(19) $$Nu=\frac{hD_{h}}{k}$$

where the temperature difference between the wall temperature and the mean fluid temperature $\Delta T=\left(T_{h}{-T}_{m}\right)$, heat flux $q=Q_{h}/A$.

(20) $$T_{m}=\left(T_{in}+T_{out}\right)/2$$

The fluctuation range of the temperature is less than 0.2 K. All data are recorded until *T_in_*, *T_out_*, *T_h_*, *P_in_*, and *P_out_* become stable. Since the measurement accuracy is approximately ±0.1 K and the minimum temperature difference *T_D_* is measured to be 33.7 K.

To discuss and compare the flow distribution between different *Re* numbers, the mass flow ratio coefficient was defined as

(21) $${MFR}_{i}=\frac{{\overset{•}{m}}_{i}}{{\overset{•}{m}}_{ave}}$$

where ${\overset{•}{m}}_{i}$ is the mass flow in the *i*-th channel and ${\overset{•}{m}}_{ave}$ is the theoretical average mass flow in the channel, assuming the perfect distribution of the flow.

To evaluate and compare the results for different geometrical modifications, a degree of flow nonuniformity s was defined as an average of absolute deviation:

(22) $$s=\frac{1}{N_{c}}\sum_{i=1}^{N_{c}}\left|MFR_{i}-MFR_{ave}\right|$$

where average mass flow ratio *MFR_ave_* is 1 by definition, and where means there is an ideal flow distribution. Based on Equation (22), a degree of maldistribution parameter [16] was defined as

(23) $$\mathrm{DM}=1-\frac{s}{s_{w}}$$

where *s_w_* is the worst possible maldistribution calculated using Equation (22) with the assumption that the whole flow goes through the first channel of the PHE. The value of *DM* changes from 0 to 1, where 1 means a uniform mass flow in every channel (no maldistribution).

In order to calculate the uncertainty, we adopt the method of Schultz and Cole [39].

The absolute uncertainty of *R* is calculated as follows:

(24) $$U_{R}={\left[\sum_{i=1}^{n}{\left(\frac{\partial R}{\partial V_{i}}U_{V_{i}}\right)}^{2}\right]}^{1/2}$$

where *U_vi_* is the absolute uncertainty of each independent parameter, and *n* is the total number of parameters.

On applying Equation (18) to Equation (21), we obtain the expression of the absolute uncertainty of h.

(25) $$U_{h}=\sqrt{{\left(U_{q}\frac{1}{\Delta T}\right)}^{2}+{\left(U_{\Delta T}\frac{-q}{\Delta T}\right)}^{2}}$$

(26) $$U_{\Delta T}=\sqrt{{\left(U_{T_{h}}\right)}^{2}+{\left(U_{T_{m}}\right)}^{2}}$$

(27) $$U_{T_{h}}=0.2\sqrt{{\left(T_{1}\right)}^{2}+{\left(T_{2}\right)}^{2}+{\left(T_{3}\right)}^{2}+{\left(T_{4}\right)}^{2}+{\left(T_{5}\right)}^{2}}$$

(28) $$U_{T_{m}}=0.5\sqrt{{\left(U_{T_{in}}\right)}^{2}+{\left(U_{out}\right)}^{2}}$$

The average relative error of the heat flux (*q*) is below 5%, and the temperature difference between *T_h_* and *T_m_* (ΔT) is slightly higher than 33 K. According to Table 3 and Equations (25)–(28), the relative uncertainty of the heat transfer coefficients ($\frac{U_{h}}{h}\times 100\%$) is less than 8.3%.

The definition of the flow velocity ($u_{m}=Q/S,S=l_{1}\times w_{1}$) can be used to obtain the absolute uncertainty of the flow velocity.

(29) $$U_{u_{m}}=\sqrt{{\left(\frac{1}{S}U_{Q}\right)}^{2}+{\left(-\frac{Q}{S^{2}}U_{S}\right)}^{2}}$$

(30) $$U_{S}=\sqrt{{\left(w_{1}U_{l_{1}}\right)}^{2}+{\left(U_{w_{1}}l_{1}\right)}^{2}}$$

(31) $$\frac{U_{u_{m}}}{u_{m}}=\sqrt{\frac{{\left(\frac{1}{S}U_{Q}\right)}^{2}+{\left(-\frac{Q}{S^{2}}U_{S}\right)}^{2}}{{\left(Q/S\right)}^{2}}}=\sqrt{{\left(\frac{1}{Q}U_{Q}\right)}^{2}+{\left(-\frac{1}{S}U_{S}\right)}^{2}}$$

According to Table 3 and Equations (29)–(31), the relative uncertainty of the calculated average velocity ($\frac{U_{u_{m}}}{u_{m}}\times 100\%$) is 3.9%.

On substituting Equation (6) into Equation (24), the relative uncertainty of the friction coefficient can be given by

(32) $$\frac{U_{f}}{f}=\sqrt{{\left(\frac{U_{\Delta P}}{\Delta P}\right)}^{2}+{\left(\frac{U_{D_{h}}}{D_{h}}\right)}^{2}+{\left(\frac{U_{L}}{L}\right)}^{2}+{\left(\frac{U_{u_{m}}}{u_{m}}\right)}^{2}+{\left(\frac{U_{\rho }}{\rho }\right)}^{2}}$$

On using Table 3 and Equation (32), the relative uncertainty friction coefficient *f* can be calculated as 9.5%.

We set the mass flow rates of 0.04 kg/s, 0.08 kg/s, 0.13 kg/s, 0.18 kg/s, and 0.22 kg/s to ensure that the inlet velocity is 0.06 m/s, 0.12 m/s, 0.18 m/s, 0.24 m/s, and 0.3 m/s, respectively. Table 4 shows the comparison of the experimental and numerical results of the heat-transfer coefficient and pressure drop under five different velocities.

## 4. Experimental Results

### 4.1. Convective Heat Transfer Coefficient and Nusselt Number

Following the discussion on the velocity, pressure, temperature, and the synergy angle distribution for the models, the simulation and experimental results are compared for the convective heat-transfer coefficient and Nusselt number. Table 4 shows the heat-transfer coefficient for M6 calculated from the exoperimantal data using Equation (18) and from the numerical results. It is observed that the results obtained from the simulation well predicts the experimental results. The obtained maximum discrepancy between the heat-transfer coefficients of the simulation and experiment is 6.4% for *Re* = 2000. Table 4 compares the results for Nusselt number obtained from the simulation and experimental results. The maximum discrepancy in the Nusselt number between the simulation and experimental values is 4.7% observed for *Re* = 2000. This indicates that the simulation results agree well with those of the experiment.

### 4.2. Pressure Drop and Friction Factor

Table 3 shows the pressure drop plots from the simulation and the experiments. There is a strong agreement between the plots showing the simulation results well predicts the experimental data with *Re* = 2000 The pressure drop measured in the experiments is slightly larger than that of the simulation results with a less than 2.8% deviation. The maximum error in the pressure drop between the simulation and experiment is 3.6% where the Reynolds number is 800. A comparative analysis of the friction factor between the experiment and numerical simulation is calculated by Equation (6). For a given Reynolds number, the maximum error of the friction factor between the simulation and experimental values is 4.3%.

Table 4 also shows the comparison of the experimental and numerical results of the heat-transfer coefficient and pressure drop under five different velocities. Comparing the experimental results and the computational results verifies the reliability of using the simulation results to develop a design scheme. Knowing this, the important heat-transfer factors are compared for all six models.

## 5. Results and Discussion

### 5.1. Simulation Results

#### 5.1.1. Grid Independency

The numerical simulation results followed by the experimental results are discussed. For the numerical simulation, M6 is selected to verify the grid independency of the numerical solution. We performed the grid-independency test system comprising 1496772, 2423946, 3422575, 4496728, and 5393368 grids. Errors between the obtained temperature and pressure drop values in the heating-surface are found to be sufficiently low to prove the grid-independency of the solution. The verification of the grid independency can be observed using the values in Table 5. To save the time and computing cost of the solution, the grid system with 3422575 grids is adopted to conduct the subsequent calculations.

Simulation are undertaken at several flow rates given in Table 2, however, the results for *Re* = 2000 are discussed here for all models as the representative results for numerical simulations. The heat-transfer parameters will be later compared with experimental results for all flow rates considered in this study.

#### 5.1.2. Simulation procedure

(1) Velocity contours at the mid plane of the channel

**Figure 5:**
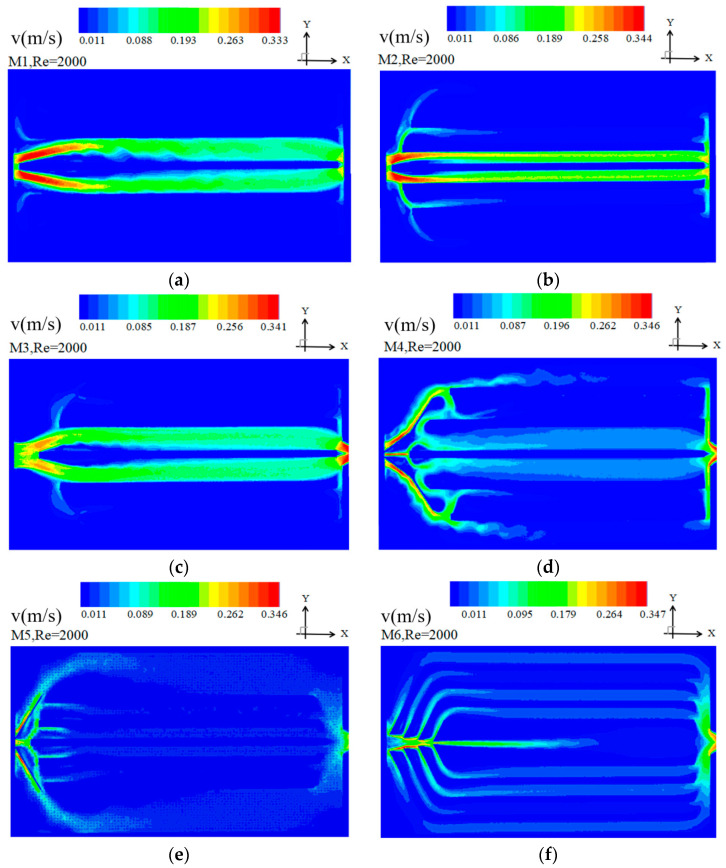
Velocity contour at the mid plane of the heat sink at *Re* = 2000.

Figure 5 shows the velocity contours for M1–M6 at *Re* = 2000. Different patterns are observed for the models. From Figure 5a,c for M1 and M3, it can be observed that the majority of the fluid flows into the two middle channels of the heat sink as the flow velocity is higher in such areas. However, there is almost no fluid in the other channels in comparison, which means that the velocity distribution of the heat sink is non-uniform for M1 and M3.

For M2, however, as shown in Figure 5b, it is visible that much more fluid has been separated to the side channels of the heat think compared to M1 and M3. This is because of the change in the width of the side channels, i.e., the width of the side channels is larger than that of the middle channel. The other factor that causes the fluid separation to the sides is altering the length of the side channels. The length of the side channels is shorter than that of the middle channel for M2. Such changes in the channel geometry make the flow resistance of the middle channel larger.

For models M4–M6, the velocity of the side channels is considerably larger than that of the side channels in M1–M3, which means the velocity distribution is obviously much more uniform in M4–M6 than in M1–M3. This is due to adding pairs of baffles in the heat sink, which widens the flow path of the middle channel leading to much more fluid flowing to the side channels. It can be observed that strong vortices in M4 are generated causes the fluid flowing into side channels. The fluid flows to the two middle channels from the side channels due to smaller pressure drop of the two middle channels.

In the case of M5, it is seen that the flow velocity accelerates toward both sides of the channel. However, it decelerates after reaching the wall in a way that the velocity in the two middle channels are still larger than that of other channels. It is clear that the velocity distribution is not uniform for this design.

In the case of M6, shown in Figure 5f, it can be observed that the velocity in all channels have closer values and the velocity distribution is nearly uniform at the entrance. It is also seen that all channels are almost equally contributing in fluid transfer. This can be explained as the effect of increasing the number of baffles which makes the resistance of the channels become nearly the same.

(2) Pressure distribution at the mid plane of the heat sink

**Figure 6:**
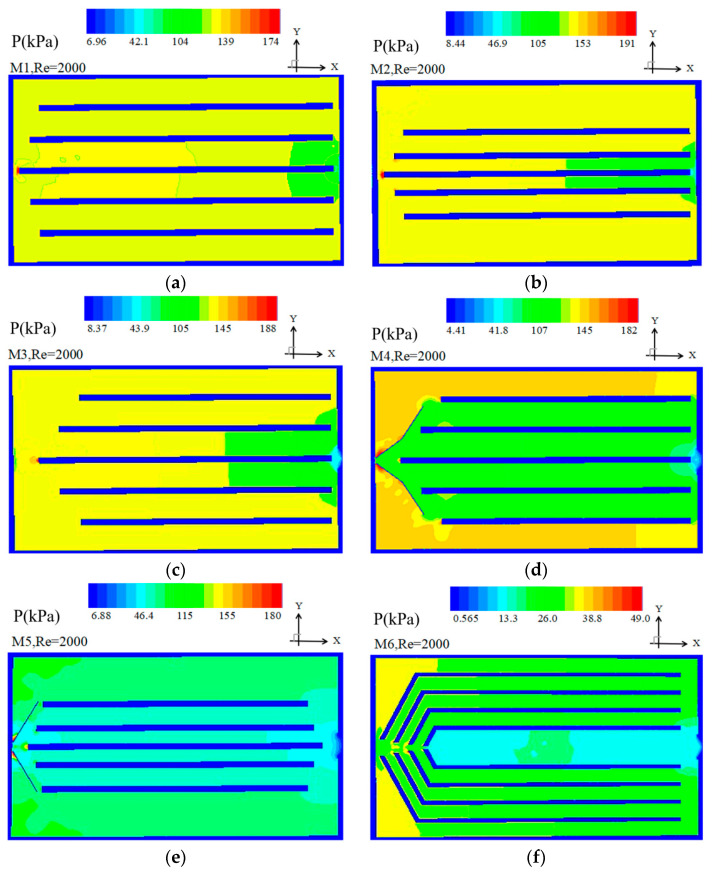
Pressure contour at the mid plane of the heat sink at *Re* = 2000.

Figure 6 shows the pressure drop distribution for M1–M6 at *Re* = 2000. From Figure 6, it can be observed that the pressure of the heat sink is totally different between the six models. It is seen from Figure 6a,c that the pressure drop of the two middle channels is lower than that of the other channels for M1-M3 leading to larger velocity of the two middle channels observed in Figure 5a–c.

In M4, the pressure drop of the marginal channel is greater than that of the four middle channels. Therefore, the velocity of the side channel in M4 is dramatically lower than the other channels (as shown in Figure 5d). The pressure drop of two middle channels are slightly lower than that of the other two middle channels leading to larger velocity of the two middle channels.

In M5 shown in Figure 6e, the pressure drop of the middle channel is slightly lower than that of other channels leading to much fluid running to the side channels as shown for the velocity distribution in Figure 5e. Figure 6f shows the pressure map for model M6. It can be observed that the pressure drop in all the channels is nearly uniform with a slightly higher pressure drop in the middle along the center line. Consequently, the velocity of all the channels in M6 is uniform in all channels and slightly higher in the centre line as shown in Figure 5f.

(3) Temperature contour at the mid plane of the heat sink

**Figure 7:**
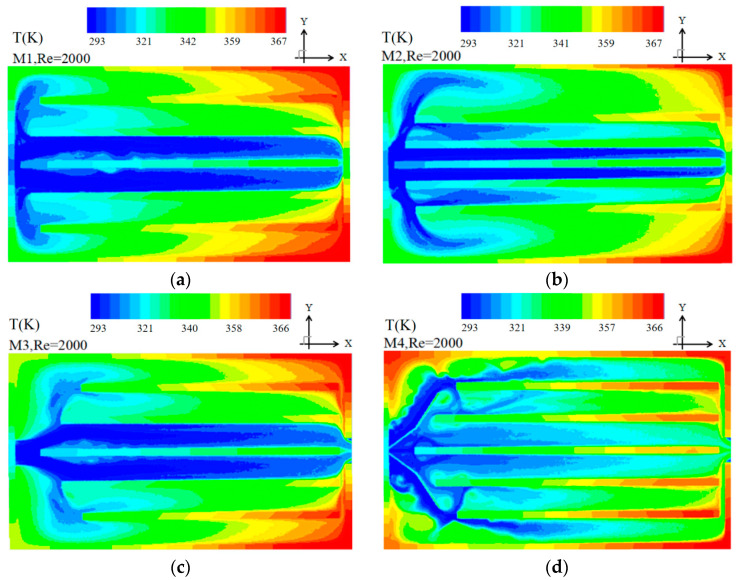

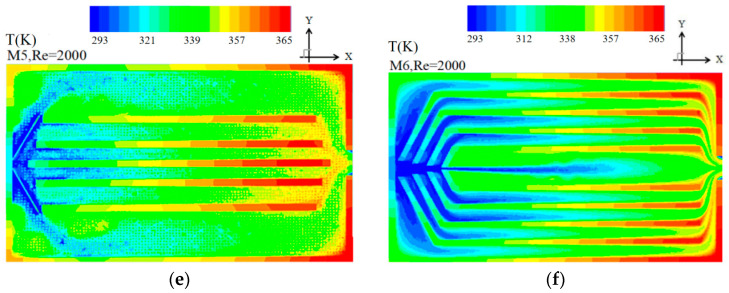
Temperature contour at the mid plane of the heat sink at *Re* = 2000.

Figure 7 shows the fluid temperature map in M1–M6 at *Re* = 2000. Temperatures vary from the lower value of 293 K to 367 K in the heat source. In M1, it is observed that the maximum fluid temperature is 367 K and the fluid temperature of the two middle channels is lower than that of the other channels, which means that the velocity of the two middle channels is too high to achieve the ideal heat transfer. It can be explained that the velocity of the side channels nearly approaches zero leading to larger velocity of the middle channels. The fluid temperature of side channels for M1–M3 is higher than other channels owing to the graded length of the channel, and thus, a small amount of fluid flows into the side channel. Compared to M1, the maximum fluid temperature for M2–M3 is 362 K and 358 K, the temperature of the channels adjacent to the two middle channels is lower than that of the side channels due to much more fluid existing. The temperature of M3 is similar to that of M1.

In M4, it can be observed that the fluid temperature of the middle channels is slightly smaller than other channels owing to the introduced pair of baffles that guide the fluid to the side channels, and the maximum fluid temperature is 347 K, which indicates the velocity uniformity is still not satisfied due to some fluid re-flowing again into the two middle channels in consequence of the lower pressure drop as shown in Figure 6d.

In M5, the maximum fluid temperature is 341 K the fluid temperature at the marginal and the channels adjacent to two middle channels are slightly higher than that of the other channels. Compared to M4, more fluid is guided to the marginal channels owing to better flow distributions (as shown in Figure 5e) leading to better temperature uniformity of the fluid. However, the fluid temperature is still uneven.

It can be observed in Figure 7 that the temperature of all the channels of M6 is more uniform than that observed with other models and the maximum fluid temperature is only 336 K owing to the connecting multi-baffle introduced leading to better flow uniformity.

(4) Temperature contour of the bottom plate of the heat sink

**Figure 8:**
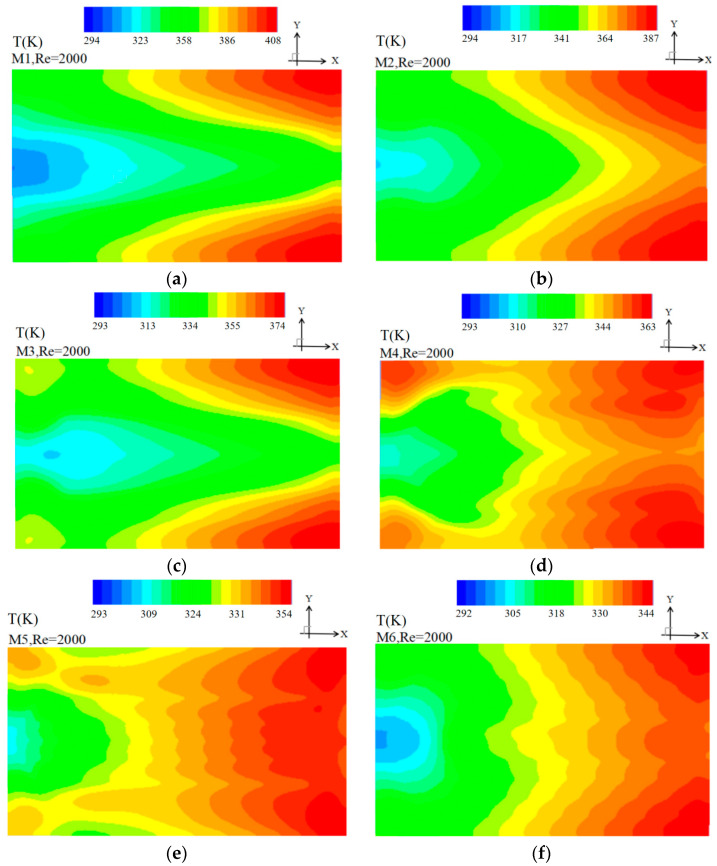
Temperature contour of the bottom plate of the MBHS at *Re* = 2000.

The multiple baffle heat sinks (MBHS) are tested and the temperatures are measured in the bottom plate. Figure 8 shows the temperature map of the bottom plate of the MBHS for all the six models having *Re* = 2000. It can be observed that the temperature difference between the maximum temperature and minimum temperature showed in color map unit for M6 is the smallest among the six models simulated. The heating-surface temperature difference is only 51.7 K for *Re* = 2000. The hot zones are observed near the outlet due to the higher fluid temperatures in consequence of the heat conduction from the heat surface. The hot spots are also observed (1) at the edges of M6 due to the absence of the channels, (2) at the side channels due to the smaller velocity of the side channels as shown in Figure 7. However, the temperature difference of M1 is 114 K and the hot zones are observed at two sides of M1 due to smaller velocity of the side channels compared to that of the middles channels. The temperature difference of M2 is 92.5 K, which indicates that M2 has a better temperature uniformity than M1. This is attributed to the more fluid flows into the side channels. The temperature difference of M3 is 80.1 K and the hot zones are similar to M1.

The temperature difference of M4 is 69.3 K due to the baffle introduced which leads to more fluid in the side channels. The hot zones are observed at the four corners due to the fluid absent. The temperature difference of M5 is 60.2 K and the hot zones are observed at the outlet including the middle and corners. Compared to M6, the hot spots of M5 are also observed at the middle channels of the outlet due to smaller velocities.

(5) Field synergy angle at the mid plane of the heat sink

**Figure 9:**
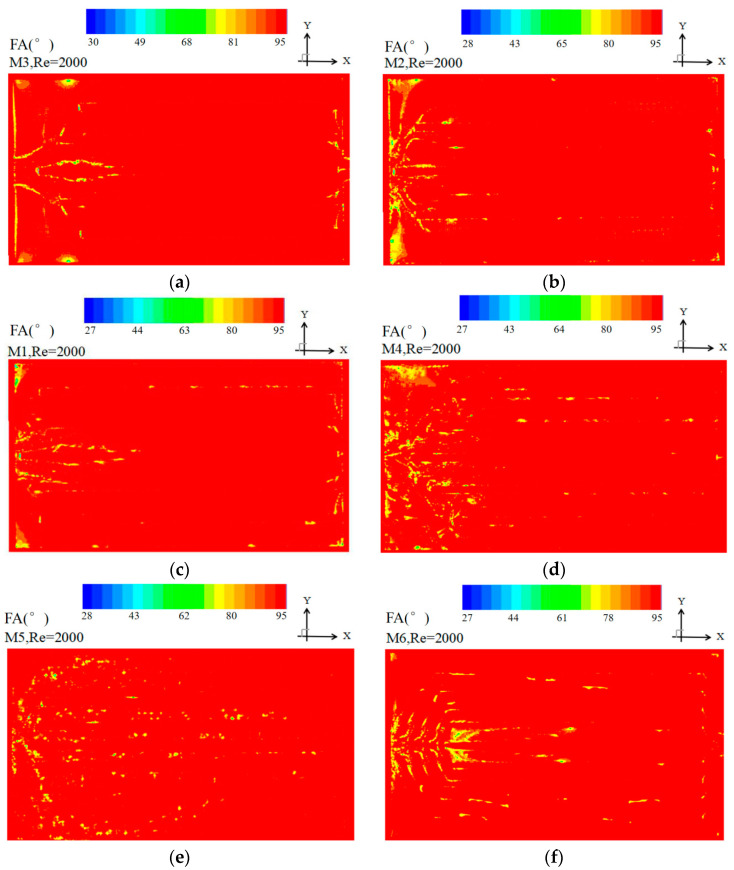
Field synergy angle contour of at the mid plane of the heat sink at *Re* = 2000.

Figure 9 shows the field synergy angle contour for M1–M6 at *Re* = 2000. In M1 and M3, it can be observed that the field synergy angle near the two middle channels is less than 90° at the entrance. It well explains the velocity contour distribution as shown in Figure 5. In M2, all the field synergy angles of each channel are smaller than 90° while those of the two middle channels are smaller than other channels. In M4–M5, it was found that the angle value of each channel is less 90° and is relatively uniform owing to the inserted pair of baffles. By introducing the multi-baffle in M6, the field synergy angle of each flow channel becomes nearly uniform and the smallest.

### 5.2. Model Comparison

(1) The temperatures in six models

**Figure 10:**
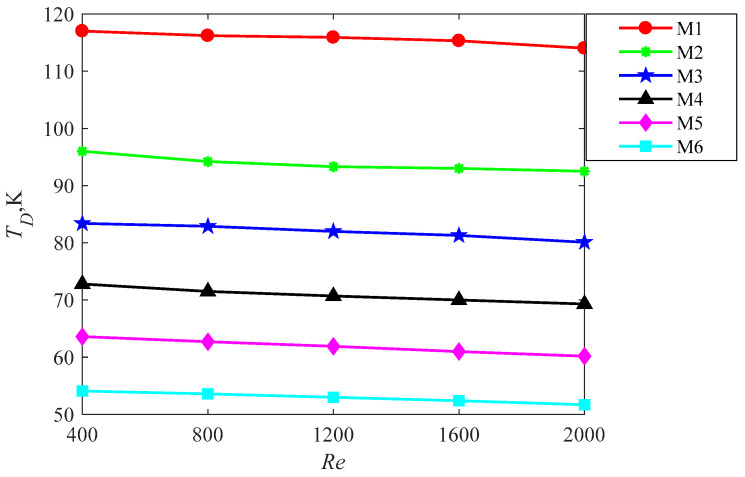
The temperature difference between the maximum and minimum temperature of the heating-surface for six models.

The temperature difference between the maximum and minimum temperature of heating-surface for six models is used as a criteria of the temperature uniformity of the heating surfaces. Since the temperature gradient of the heat sink is still in challenge, this paper aims to reduce the temperature gradient by devising new configurations for the heat sink. The smaller value of the temperature difference, the better the temperature uniformity. The effects of the different models on the temperature difference between maximum temperature and minimum temperature of the heating surface in M1–M6 is shown in Figure 10. It is observed the temperature difference decreases with the increase of the Reynolds number for all the six models due to a higher heat transfer rate received leading to the lower average temperature of the heating surface. For a fixed Reynolds number, the temperature difference of M6 is the lowest due to the excellent velocity uniformity obtained by the multi-baffle as shown in Figure 5f. The lowest temperature of the heating-surface is achieved for M6 as it can be seen in Figure 10, which indicates the highest heat transfer rate. Therefore, M6 possesses the lowest temperature difference that is associated with the best temperature uniformity.

(2) The heat-transfer performance for six models

**Figure 11:**
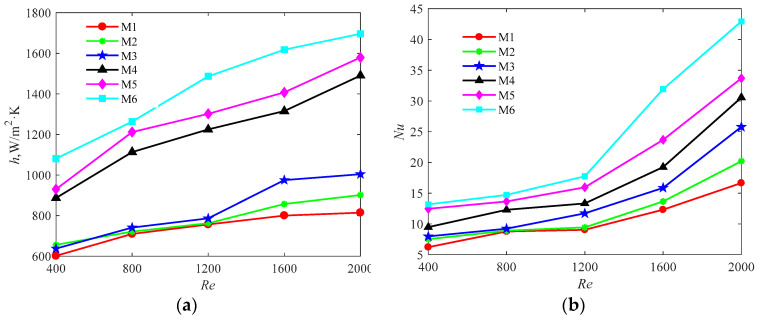
The heat-transfer performance for six models, (**a**) the heat-transfer coefficient; (**b**) Nusselt number.

The comparison of the heat-transfer coefficient and Nusselt number of the six models in the simulation is illustrated in Figure 11a,b. With the increase of Reynolds number, both the heat-transfer coefficient and Nusselt number increase. For all Reynolds numbers, the heat-transfer coefficient and Nusselt number of M6 are the largest, which indicates the acceptable heat-transfer performance of M6 due to the lower temperature of the heating surface (as shown in Figure 8) and higher outlet fluid temperature comparing other models. The maximum convective heat transfer of M6 reaches a value of 1674.85 W/m^2^·K and the Nusselt number is 37.5 obtained at the highest evaluated flow rate at *Re* = 2000. These values are the lowest for M1 with the heat-transfer coefficient of 814.84 W/m^2^·K and the corresponding Nusselt number of 16.66.

(3) Friction factor for six models

**Figure 12:**
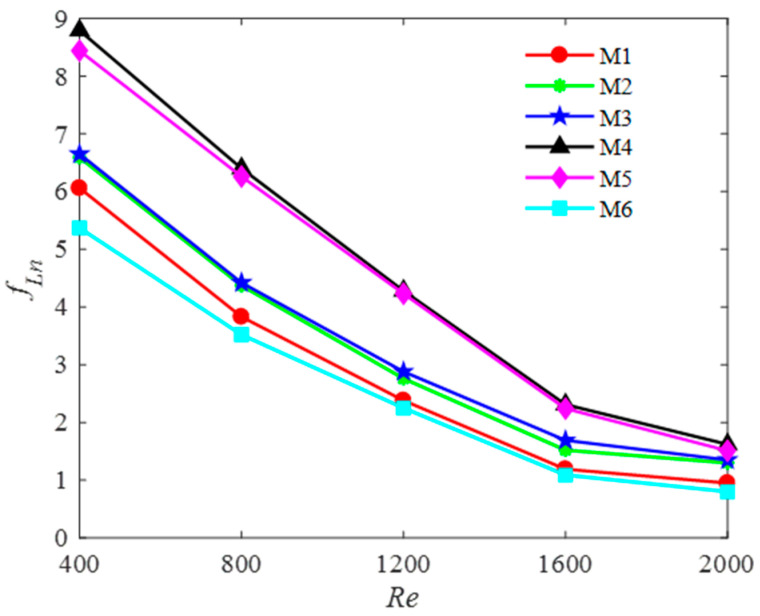
Pressure-drop and friction factor for six models.

The variations in the friction factor with the Reynolds number are shown in Figure 12. It is observed from Figure 12 that the friction factor decreases gradually as the Reynolds number increases. Compared to M4 and M5, the friction factors for M1–M3 are smaller as no baffles are introduced. However, owing to the presence of a pair of baffles in M4–M5, the flow path is enlarged such that the value of the friction factor becomes larger at *Re* = 2000. However, the value of the friction factor of M6 is only 0.81 at *Re* = 2000 owing to the introduced multi-baffle. It also can be found that the pressure drop values have an increasing trend with increasing Reynolds number. The pressure drop in M4 is the largest because stronger vortices are generated by the two longest baffles (as discussed for the results shown in Figure 6). It indicates that the fluid is guided from the core flow region to the side channel, which increases the flow path that leads to a larger pressure drop. Compared to M6, the pressure in M5 is smaller than that in M4 owing to the smaller scale and weaker intensity of the vortices in consequence of the shorter baffles added. After adding the multi-baffle, the pressure drop in M6 becomes optimal and the pressure drop from the inlet parallel channels to the outlet of the parallel channels is only 28.8 kPa when the Reynolds number equals to 2000.

(4) Influence of Reynolds number on average field synergy angle

**Figure 13:**
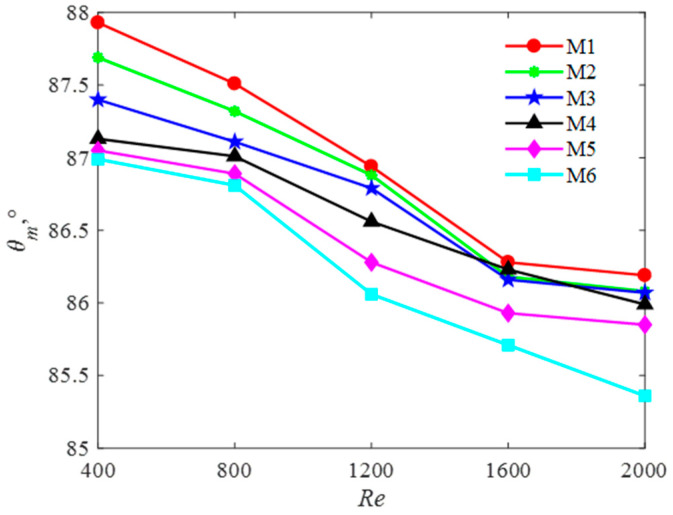
The influence on the average field synergy angle for six models.

The effects of the different models on the average field synergy angle are shown in Figure 13. As the Reynolds number increases, the value of the field synergy angle decreases. For a fixed *Re*, the average field synergy angle of M6 is the smallest due to multi-baffle added. The maximum average field synergy angle of M1 is 86.21° when the Reynolds number equals to 2000, and the minimum average field synergy angle of M6 is just 85.33°. The field synergy angle of M6 at *Re* = 2000 is smaller than that of *Re* = 400. It can be concluded that an optimal multi-baffle presented in M6 can reduce the value of the average field synergy angle.

(5) Performance evaluation

**Figure 14:**
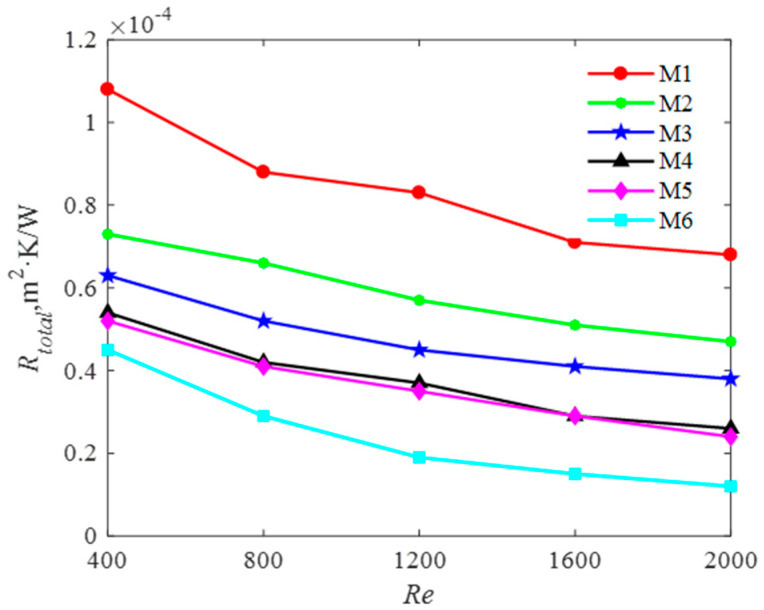
Total thermal resistance for six models.

Total thermal resistance is a ratio of the temperature difference to the heat flux. Performance Evaluation Criterion is employed to measure the total resistance for heat-transfer [40]. Therefore, a lower value of the total thermal resistance may be good for heat-transfer. Figure 14 examines the variation of the total resistance with the Reynolds number for the six models simulated. For all *Re*, it is seen that the total thermal resistance of M6 is the lowest comparing other five models. The total thermal resistance of the heat sink is comprised by conductive resistance and convective resistance. The conductive resistance is related to the distance between the heating surface and channels, and the heat conductivity of the heat sink. Therefore, the conductive resistance is independent of Reynolds number. However, the convective resistance decreases with the increase Reynolds number due to higher heat-transfer coefficient obtained. Therefore, for all the six models, the conductive resistance is the same value in spite of the convective resistance is different. The convective resistance of M6 is the lowest due to the highest coefficient as shown in Figure 14.

The evaluation of the overall thermal performance of adding multi-baffle inserted into heat sink was calculated using the Performance Evaluation Criterion proposed by Webb [41] in 1981. The *PEC* is defined here as:

(33) $$PEC=\frac{\left(Nu/Nu_{0}\right)}{{\left(f/f_{0}\right)}^{\frac{1}{3}}}$$

where *Nu*_0_ and *f*_0_ denotes the Nusselt number and friction factor of the plain channel, respectively.

**Figure 15:**
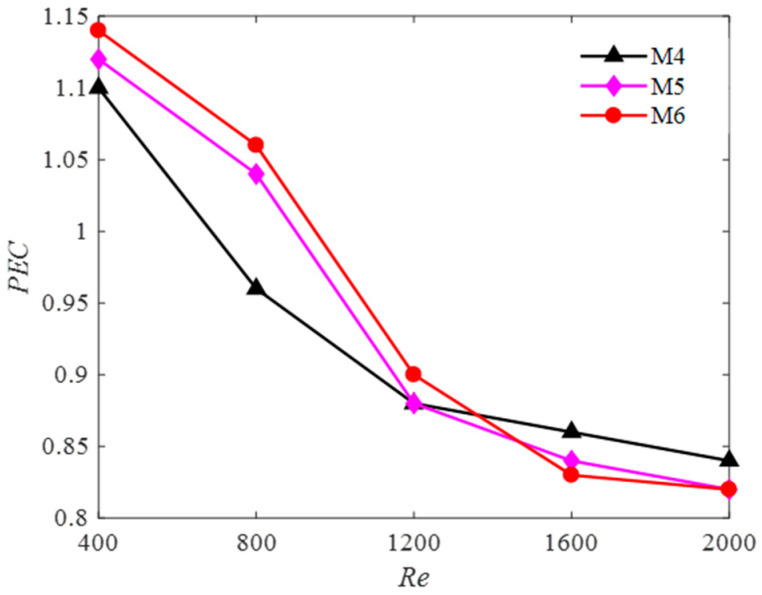
The effect of different models on Performance Evaluation Criterion (*PEC*).

Figure 15 represents the influence of different models on the over all heat-transfer performance of the heat sink for various Reynolds numbers. It can be seen apparently that the *PEC* for M6 has a best performance in low Reynolds number range, and the value of the *PEC* is larger than 1. The *PEC* values for M4 are higher than other models in the high Reynolds number range, however, it remains less than 1 for the high Reynolds number range considered. Above all, M6 is suggested as the best type of heat sink.

### 5.3. Model Selection and Limits

Table 6 and Table 7 show the improvement in each factor value using model M6 comparing other models and the best uniformity of six models. The minimum and maximum improvements are also shown in the table. It is seen that the model M6 improves all factors significantly. Therefore, a design based on M6 model is suggested for the applications require maximizing flow uniformity and heat-transfer performance.

## 6. Conclusions

In this paper, a new type of multi-baffle-type heat sink is presented. The flow and heat-transfer performances of the multi-baffle-type heat sinks with different inlet velocities through experiments and numerical simulations are investigated. The obtained results indicate that the equivalent heat transfer of M6 can reach a value of 1758.59 W/m^2^·K and the temperature difference between the maximum and minimum temperature of the heating surface is 51.7 K with a pressure drop of 29.6 × 10^3^ Pa at *Re* = 2000. The simulation results show that M6 has the best heat-transfer performance, flow-evenness performance, and temperature uniformity performance comparing the models investigated in this research. By setting up an experimental platform, the simulation results of M6 are verified. The following conclusions can be drawn:

(1) As compared with the five models M1–M5, the velocity field of M6 is more uniformly distributed without the aid of external electronic devices.
(2) M6 takes the advantage of dispersing heat from the high-temperature zone in a well-proportioned way.
(3) The heat-transfer and flow performance of the heat sink can be effectively improved by employing optimally shaped baffles.
(4) The average synergistic field angle decreases with the increase of the multi-baffle.
(5) The heat-transfer coefficients and pressure drop of the experimental and simulation results are consistent with each other, which verifies the correctness of the numerical method and results.
(6) Among six models, M6 possesses the lowest total thermal resistance.

## Figures and Tables

**Table 1:** Geometric parameters of multi-baffle-type heat sink (MBHS).

Model	L/W/H (mm) H_1_/L_*in*_/L_*out*_	L_*n*_ (mm) (*n* = 1, 2, 3, 4)	W_*n*_ (mm) (*n* = 1, 2, 3, 4, 5)	L_0*n*_ (mm) (*n* = 1, 2, 3, 4)	*α*/*β* (°)
M1	214/139/5	198/191/185	17.8/17.8/17.8	--	--
	3/18/18				
M2	214/139/5	198/191/185	9/13/31.4	--	--
	3/18/18				
M3	214/139/5	186/173/160	17.8/17.8/17.8	--	--
	3/18/18				
M4	214/139/5	186/173/160	17.8/17.8/17.8	22/28	80° 120°
	3/18/18				
M5	214/139/5	186/173/168	9/13/31.4	34	120°
	3/18/18				
M6	214/139/5	170/166 162/158	15.5/13.6 12.6/11.7	16/30 42/54	115° 30°
	3/18/18				

**Table 2:** Reynolds numbers corresponding to the heat sink inlet fluid velocities (*u*_1_).

*u*_1_ (m/s)	0.06	0.12	0.18	0.24	0.3
*Re*	400	800	1200	1600	2000

**Table 3:** Uncertainties of the experimental parameters.

Parameter	Absolute Uncertainty	Relative Uncertainty
Slot width (*l*_1_)	±0.01 mm	
Slot depth (*w*_1_)	±0.01 mm	
Temperature	±1 K	
Pressure		±2%
Liquid flow rate		±1%
Power		±2%
Heat loss of the heat unit		4.1%
Heat transfer coefficient		8.3%
Average velocity		3.9%
Friction coefficient		9.5%

**Table 4:** Measured (subscribed with exp) and simulated (subscribed with sim) equivalent heat-transfer coeffcients and pressure drop with different velocities for M6.

*V* (m/s)	Δ*T_exp_* (K)	Δ*T_sim_* (K)	Δ*T* Error	Δ*P_exp_* (Pa)	Δ*P_sim_* (Pa)	Δ*P* Error	*h_exp_* (W/m^2^·K)	*h_sim_* (W/m^2^·K)	*h* Error	*Nu_exp_*	*Nu_sim_*	*Nu* Error
0.06	118.2	115.4	2.4%	5220	5110	1.3%	725.42	681.15	6.1%	13.3	12.9	3.0%
0.12	83.5	78.0	6.6%	7700	7450	3.6%	1349.62	1263.31	6.4%	14.8	14.1	4.7%
0.18	53.8	49.5	8.0%	11300	11260	0.5%	1612.04	1601.53	0.7%	15.9	15.5	2.5%
0.24	46.5	43.1	7.3%	17900	17300	3.4%	1706.87	1623.32	4.9%	29.4	28.8	2.0%
0.3	33.7	31.8	5.6%	29650	28800	2.9%	1758.59	1674.85	6.3%	38.2	37.5	1.8%

**Table 5:** Grid independence test results.

Grid Number	Δ*P* (KPa)	Relative Error	Δ*T* (K)	Relative Error
1496772	29.63	0.0082%	372.33	0.0072%
2423946	29.63	0.0064%	372.35	0.0072%
3422575	29.64	-------	372.37	-------
4496728	29.65	0.0023%	372.37	-------
5393368	29.66	0.0023%	372.35	0.0037%

**Table 6:** The improvement value in field synergy angle, temperature difference, equivalent heat-transfer coefficient, Nusselt number, pressure drop and friction factor of M6.

Factor	*θ*°	Δ*T* (K)	*h* (W/m^2^·K)	*Nu*	Δ*P* (kPa)	*f*
M6	85.33	51.7	1758.59	42.6	29.6	0.82
Improvements	min	min	max	max	max	min

**Table 7:** Values of the *DM* coefficients for M1–M6 at *Re* = 2000.

Model	M1	M2	M3	M4	M5	M6
*DM*	0.591	0.669	0.763	0.767	0.882	0.915

## Nomenclature

*A_c_*	cross sectional area, m^2^	*T_b,avg_*	average channel base temperature, K
*DM*	degree of maldistribution	∇T	temperature gradient
*D_h_*	hydraulic diameter, m	Δ*T*	average temperature difference, K
*f*	friction factor	*T_m_*	average fluid temperature, K
*f*_0_	friction factor of a plain channel	*T_in_*	inlet temperature, K
*H*	total height of cooling plate, mm	*T_out_*	outlet temperature, K
*H*_1_	height of the counter sink, mm	*T_h_*	average temperature of heating surface, K
*h_conv_*	convective heat transfer coefficient, W/m^2^ K	*T_D_*	temperature difference between the maximum and minimum temperature of heating surface, K
*k_s_*	thermal conductivity of Al, W/m K	*u*_1_	axial velocity, m/s
*K_f_*	thermal conductivity of fluid, W/m K	*u_m_*	mean axial velocity, m/s
*L*	length of heat sink, mm	∇V	velocity vector
*L*_i_	length of flow channel, mm	*W_i_*	width of flow channel, mm
*L*_0i_	length of baffle, mm	*x_i_*	rectangular coordinates
*MFR*	mass flow ratio coefficient	**Greek symbols**	
Nu	Nusselt number	μ	kinematic viscosity, kg/ms
*Nu*_0_	Nusselt number of a plain channel	ρ	density, kg/m^3^
*p*	wetting perimeter, m	$\theta_{m}$	average field synergy angle, °
Δ*P*	pressure drop of heat sink, Pa	β	baffles intersection angle, °
*P_out_*	outlet pressure, Pa	ζ	head loss coefficient
*P_in_*	inlet pressure, Pa	**Subscripts**	
*q*	heat flux, W/m^2^	*ave*	average
*q_m_*	mass flow rate of liquid, kg/s	*i*	index in x-direction
*Re*	Reynolds number	*j*	index in y-direction
*R_total_*	total thermal resistance, m^2^·K/W	*n*	flow channel number
*s*	average absolute deviation	*w*	heating surface
*T*	temperature, K	*x*,*y*,*z*	three coordinates shown in Figure 1, mm
